# Serum Cytokine of IL-10 and IL-12 in Chronic Liver Disease: The Immune and Inflammatory Response

**DOI:** 10.1155/2015/707254

**Published:** 2015-12-10

**Authors:** Hoda Mohamed El-Emshaty, Wesam Ahmad Nasif, Ibrahim Eldsoky Mohamed

**Affiliations:** ^1^Gastroenterology Laboratories, Gastroenterology Surgical Center, Mansoura University, Mansoura 35516, Egypt; ^2^Molecular Biology Department, Genetic Engineering and Biotechnology Research Institute, Sadat University, Sadat City, Egypt; ^3^Biochemistry Department, Faculty of Medicine, Umm Al-Qura University, Makkah, Saudi Arabia; ^4^Pathology Department, Faculty of Medicine, Mansoura University, Mansoura 35516, Egypt

## Abstract

The current study was designed to investigate the potential association of serum interleukin-10 and interleukin-12 with HCV infection in chronic liver disease and to evaluate their possible role as new biomarkers in HCC development.* Material and Methods*. Forty-one patients suffering from chronic liver disease (33 patients harbor HCV infection and 8 are HCV-negative patients) were enrolled in the present study and histopathologically diagnosed into 15 patients with HCC, 16 patients with LC, and 10 patients with liver histology compatible with precirrhotic hepatitis (PCH). Ten patients complaining of cholecystitis were included as nondisease control. Serum levels of IL-10 and IL-12 were measured by enzyme linked immunosorbent assay (ELISA).* Results*. HCV-infected patients showed elevated expression of IL-10 and IL-12 compared to nondisease controls (*P* < 0.0001) but there is no significant difference with respect to their expression in HCV-negative patients. Serum IL-10 and IL-12 were elevated significantly with disease progression (*P* < 0.0001) and a positive correlation coefficient was detected between IL-10, IL-12 (*r* = 0.785, *P* < 0.0001), and transaminase values suggesting their possible role in chronic inflammation progression leading to HCC.* Conclusion*. IL-10 and IL-12 might be involved in chronic inflammation progression leading to HCC and their evaluation could be used as new biomarkers to reflect the degree of inflammation in HCC development.

## 1. Introduction

Hepatitis C virus (HCV) infection affects almost 3% of the world's population with the highest prevalence (15%) in Egypt [1]. HCV infection results in chronic hepatitis in more than 70% of infected patients, while 20–30% of patients recover spontaneously [2]. The pathogenesis and outcome of viral infections are significantly influenced by the host immune response. The immune system is able to eliminate many viruses in the acute phase of infection. However, some viruses, like hepatitis C virus (HCV) and hepatitis B virus (HBV), can evade the host immune responses and establish a persistent infection [3].

The precise role of the immune response in patients with HCV infection, in particular the relationship between the levels of inflammatory/regulatory cytokines and the course of HCV infection, is still unclear. Recent work has suggested that these cytokines can trigger distinct patterns of protective or immunopathological responses and that they are involved in the clearance or establishment of chronic HCV infection [4]. However, the balance of proinflammatory and regulatory cytokines appears to be important in determining the course of HCV infection [5].

Cytokines play an important role in differentiation, maturation, and functional activation of immune cells [6]. Cytokines are produced by multiple cell types such as NK cells and macrophages CD4^+^T cells and CD8^+^T cells. Responses are referred to as Th1-like and Th2-like after the original description of the cytokine profiles produced by subsets of CD4^+^T cells [7]. Th1-like responses include IL-2, TNF-*α*, and IFN-*γ* secretion and are required for generation of cytotoxic T lymphocytes and NK cell activation during the host antiviral immune response. Th2-like responses produce IL-4 and IL-10, which help augment antibody production and inhibit development of the Th1 response [8].

IL-10 is a pleiotropic cytokine produced by macrophages T-helper 2 (Th2) cells and B-lymphocytes, and both can stimulate and suppress the immune response. IL-10 has been shown to inhibit various immune reactions [9]. Interleukin-12 (IL-12) is one of the most important proinflammatory cytokines produced mainly by antigen presenting cells as a result of IFN-*γ* stimulation [10] and presented with the initiation of immune response, so IL-12 can be considered as one of the most clearly defined factors determining Th1 and Th2 differentiation [11]. Therefore, the current study was designed to investigate serum expression of inflammatory and immunoregulatory cytokines (IL-10/IL-12) with HCV infection in chronic liver disease and to evaluate their possible role as new biomarkers in chronic inflammation progression leading to HCC.

## 2. Material and Methods

### 2.1. Study Population

Forty-one (34 males and 7 females; mean age 47.58 ± 12.42 yrs) patients suffering from chronic liver diseases were enrolled in the present study; 33 (80.5%) patients had positive reactivity for HCV infection with detectable HCV RNA and with no serological evidence of coinfection with other hepatotropic viruses or human immunodeficiency virus and 8 (19.5%) patients had negative reactivity for HCV infection. All patients gave written informed consent at gastroenterology surgical center, Mansoura University, Egypt. The studied group was subjected to clinical examination, imaging radiology, laboratory investigation, and liver biopsy. Liver tissue and blood samples of all individuals were collected and sera were stored at −70°C until being used.

Liver tissue specimens of all cases were routinely processed for histopathological diagnosis, fixed in 10% neutral buffered formalin, embedded in paraffin, and cut into 3–5 *μ*m thick sections. The studied group was histopathologically diagnosed and categorized into 15 (36.58%) patients (13 male and 2 female; mean age 57.06 ± 12.58 yrs) with HCC; 16 (39.0%) patients (12 male and 4 female; mean age 46.06 ± 7.9 yrs) with liver cirrhosis; and 10 (24.4%) patients (9 male and 1 female; mean age 35.8 ± 5.22 yrs) with liver histology compatible with precirrhotic hepatitis (Metavir score A2F3). Tumor grading of HCC was histopathologically assessed as grade I in 2 (13.3%), grade II in 4 (26.7%), and grade III in 9 (60%) of 15 HCC cases.

Also, ten (10) patients complaining of cholecystitis without a clinical history of hepatitis and without symptoms or signs of liver disease were included as nondisease control group (7 male and 3 female; mean age 35.6 ± 3.1 yrs) (Table 1).

#### 2.1.1. Biochemical and Virological Determination

Liver biochemistries including albumin, globulin, ALT, AST, alkaline phosphatase, and total bilirubin were measured by an autoanalyzer. Serological diagnosis of HBV and HCV was performed by automated immunoassay. HBsAg was measured by a commercial immunoassay kit (Abbott Laboratories, USA). Serum HCV Ab was analyzed by third-generation ELISA kit (BIOKIT, S.A., Barcelona, Spain).

#### 2.1.2. Detection of Genomic HCV RNA Strands by RT-PCR

Total HCV RNA was isolated from 125 *μ*L of the serum and purified by using SV Total RNA isolation system (Promega Co., USA). Qualitative detection of the genomic HCV RNA was done using a strand specific real-time RT-PCR with the use of thermostable enzyme (Tth) for the synthesis of cDNA at a high temperature. For the amplification of the genomic HCV RNA strand, two sets of oligonucleotide primers (Bio-Synthesis, USA) deduced from the highly conserved 5′-noncoding region of HCV genome were induced in the first and second stage, respectively.

*1st Stage*

 Sense C196 (5′-CCATGGCGTTAGTATGAGTG-3′).
 Antisense Seq-CR (5′-TGCTCATGGTGCACGGTCTA-3′).

*2nd Stage*

 Sense Seq-3 (5′-AGAGCCATAGTGGTCTGCGG-3′).
 Antisense Seq-4 (5′-CTTTCGCGACCCAACACTAC-3′).

#### 2.1.3. Enzyme Linked Immunosorbent Assays for Il-10 and IL-12

Serum levels of IL-10 and IL-12 were measured by ELISA kit (*Diaclone Research, Besancon, France*) according to the manufacturer's instruction which can detect up to 3 pg/mL of human IL-10 and 5 pg/mL of human IL-12. Briefly, 50 *μ*L of standards and samples were added in duplicate to the precoated strip well plate; then, 50 *μ*L of biotinylated antibody was added to each well as a conjugated antibody for 2 hrs. After washing three times, 100 *μ*L of streptavidin horseradish peroxidase (HRP) was added to each well for 30 min followed by 100 *μ*L of TMB substrate solution for 30 min. Finally, 100 *μ*L of stop solution was added to each well and the absorbance of the plate was detected on a plate reader at 450 nm. All incubations and recording were done at room temperature.

### 2.2. Statistical Analysis

Results were expressed as mean ± SD (median). The differences in mean were assessed by Kruskal Wallis (Mann-Whitney* U* test) and all differences were considered to be significant at *P* ≤ 0.05. Correlations were calculated by Spearman's correlation coefficient.

## 3. Results

Clinical, virological, and biochemical characteristics of the studied group were listed according to the pathological diagnosis in Table 1. Mean serum level of ALT, AST, total protein, and S. bilirubin and prothrombin time showed elevated expression with disease progression. Only ALT and AST were elevated significantly among disease groups. The differences in ALT were recorded between precirrhotic hepatitis (PCH) and other groups (with LC *P* = 0.027 and with HCC *P* = 0.003) and also the differences in AST levels were recorded between PCH and other groups (with LC *P* = 0.014 and with HCC *P* = 0.02). On contrary, serum albumin level was decreased with liver disease state but with no significant difference.

The severity of HCC was determined through a combination of clinical and laboratory evaluation within the context of Child-Pugh scoring system. Child score was recorded in HCC patients as Child A in 20% (3/15), Child B in 40% (6/15), and Child C in 40% (6/15). Serum expression of IL-10 and IL-12 according to the pathological diagnosis was listed in Table 2. IL-10 and IL-12 showed significant elevation with disease progression and the highest expression was detected in HCC compared to LC and PCH (*P* < 0.0001) but there is no significant difference between LC and PCH with respect to IL-12. However, serum level of Il-10 and IL-12 showed no significant difference with respect to tumor site, tumor grade, or Child score.

**Figure 1:**
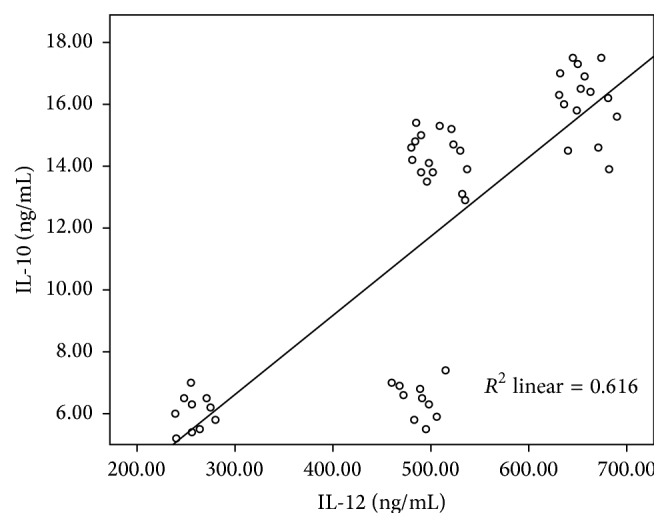
Scatter plot of IL-10 and IL-12 in patients with different pathological diagnosis (*r* = 0.785, *P* < 0.0001).

**Figure 2:**
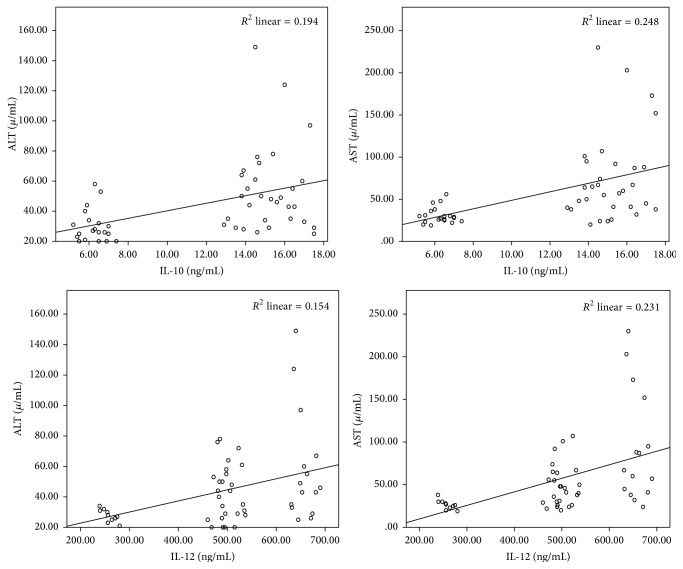
Serum levels of IL-10 and Il-12 showed significant correlation with transaminases in patients with chronic liver disease.

IL-10 showed a significant positive correlation with IL-12 in patients with different liver pathologies (*r* = 0.785, *P* < 0.0001) (Figure 1) and a significant correlation coefficient was detected between IL-10 and IL-12 with transaminase values of patients with chronic liver disease (Figure 2). IL-10 showed significant positive correlation with ALT (*r* = 0.441, *P* = 0.001) and AST value (*r* = 0.498, *P* < 0.0001). Also, Il-12 showed a significant positive correlation with ALT (*r* = 0.39, *P* = 0.004) and AST value (*r* = 0.48, *P* < 0.0001).

**Figure 3:**
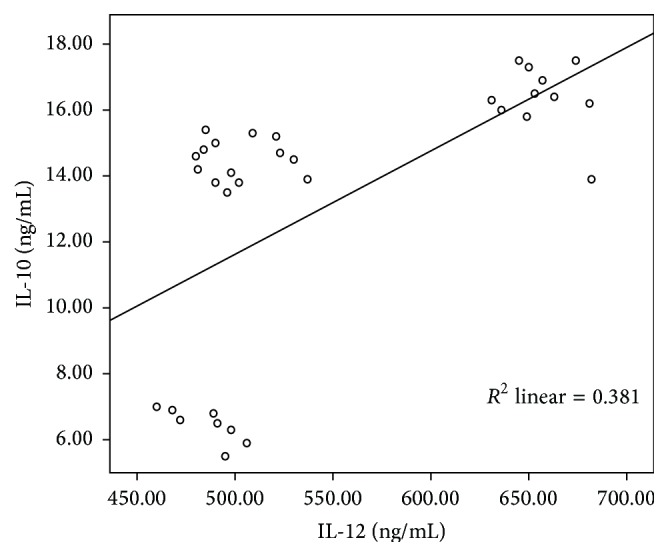
Scatter plot of IL-10 and IL-12 in patients with HCV-chronic liver disease (*r* = 0.617, *P* < 0.0001).

IL-10 and IL-12 were elevated significantly (*P* < 0.0001) in the sera of patients with HCV infection compared to nondisease control group (Table 3) and the level was elevated significantly among disease groups (*P* < 0.0001). HCV-infected patients showed IL-10 at higher level than in HCV-negative patients and in opposite to IL-12 expression pattern in HCV-positive and HCV-negative cases. The difference in cytokine expression level was low between HCV-positive and HCV-negative cases and did not differ significantly between them. Furthermore, a significant positive correlation (*r* = 0.617, *P* < 0.0001) was detected between IL-10 and IL-12 (Figure 3) in HCV-infected patients.

## 4. Discussion

Cytokines play an important role in viral clearance, infection control, inflammation, regeneration, and fibrosis and also are implicated in the pathological processes occurring in the liver during viral infection [12]. Therefore, our study was designed to investigate serum level of IL-10 and IL-12 in chronic liver disease and their association with HCV infection and to evaluate their possible role as new biomarkers in chronic inflammation progression leading to HCC.

IL-12 is one of the most important proinflammatory cytokines presented with the initiation of immune response, determining Th1 and Th2 differentiation [1]. Capone et al. [13] compared the serum level of numerous cytokines, chemokines, and growth factors in HC and LC patients with respect to those in HCC patients tested in their study. They found that the mean concentrations of all of these molecules were higher in HCC patients than in those with LC. Our results are in consistence with these findings where serum levels of IL-12 was more elevated in HCC patients than in those with LC and PCH (*P* < 0.0001) and the elevation was increased with disease progression. This suggests that the expression of these proinflammatory molecules tends to increase in the chronic inflammation progression that leads to LC and HCC and thus, their evaluation could be used for prognostic studies [12]. In spite of controversial data in the literature, several reports described that the serum level of IL-12 was significantly higher in their patients with chronic HCV infection than in healthy donors [14, 15]. In current study, serum expression of IL-12 in HCV-chronic liver disease showed significant elevation compared to nondisease individuals and the level was enhanced with disease progression suggesting that a strong proinflammatory cytokine response could play an important role in the development of hepatic injury in patients with chronic hepatitis C, and therefore, apart from contributing to viral clearance, this polarized immunological profile may contribute to the pathogenesis of liver disease [16].

Serum IL-10 concentration has been reported to be significantly elevated in patients with chronic HCV and IL-10 may be related to hepatocarcinogenesis with suppression of immune surveillance [17]. The measurement of IL-10 concentrations in serum samples of patients with chronic HCV infection by enzyme linked immunosorbent assay (ELISA) has showed contrasting results. Kakumu et al. [14] demonstrated greater spontaneous IL-10 production by peripheral blood mononuclear cells (PBMc) in patients with CHC and liver cirrhosis than in healthy controls and its decrease during IFN treatment. On the contrary, Yamashiki et al. [18] reported that the level of IL-10 in the monocyte/macrophage supernatants of patients with CHC was significantly lower than in healthy controls. In consistence with Othman et al. [19] and Hattori et al. [20], IL-10 concentration was elevated in patients with HCV, cirrhosis, and HCC and the concentrations are associated with disease progression indicating that IL-10 reflects the degree of inflammation in the liver and may be related to the development of HCC. However, increased circulating IL-10 has been reported in patients with different types of tumors including resectable HCC [21].

These results may be explained on the basis that the high serum IL-10 levels in patients with HCC result from the secretion of IL-10 by tumor cells, in addition to the production at the site of inflammatory changes with activated infiltrating mononuclear cells in the liver [22]. The immunosuppressive effects of IL-10 may play a major role in the development of neoplastic process by suppressing macrophage activation and interferon-gamma production, thereby crippling two potential mediators of an antitumor response; this may help the tumor cells escape host immune surveillance and potentate tumor cells to metastasize [19]. Also, the functional consequences of IL-10 binding to its receptors on tumor cells could be the prevention of programmed cell death and the promotion of proliferation [23]. Therefore, it has been proposed that IL-10 plays a key role in the oncogenetic and metastatic ability of neoplasms [24].

Analysis of the sequential serum data reported by Wu et al. [25] indicated a significant correlation between Il-10 and IL-12 in tolerance phase (correlation coefficient 0.42, *P* < 0.0001), suggesting that IL-10 may be bifunctional during the course of HBV infection and that its role may depend on serum levels and cooperative cytokines like IL-12 and downstream IL-2. In current study, a significant positive correlation (*r* = 0.617, *P* < 0.0001) was detected between IL-10 and IL-12 in HCV-chronic liver disease patients. IL-10 was elevated in patients with HCV infection compared to HCV-negative patients and in contrary to IL-12 expression pattern in HCV-positive and HCV-negative cases. These results suggest that elevation of serum IL-10 might be involved in downregulation of the inflammatory response in chronic liver disease [14]. Furthermore, interleukin-12 production by DC appears to be downregulated by IL-10, while with maturation, the DC can produce large amounts of IL-12 and become resistant to the suppressive effects of antigen presenting function by IL-10 [26].

In patients with chronic inflammation, IL-1*α*, IL-2R, MIF, and *β*-NGF showed significant correlation and a positive correlation coefficient with the transaminase values, which were higher in these patients than in healthy controls. Therefore, these proteins could be considered to be an index of immune activation. In particular, these results were in agreement with literature data reporting that IL-1 and IL-2R participate in the progression from liver injury to fibrosis [27, 28] and that *β*-VGF is involved in liver cancer growth and metastasis and can be used as an index of chronic infection leading to LC and HCC [29, 30]. In agreement with these studies, a significant positive correlation was detected between IL-10 and IL-12 in our study and a significant correlation coefficient was detected with the transaminase levels which were higher in all liver pathologies than in nondisease controls; the elevation was significantly recorded with disease progression suggesting that IL-10 and IL-12 could be used as an index to reflect the degree of inflammation in the liver with ultimate development of HCC. However, Liu et al. [31] reported that the levels of TNF-alpha, IL-1, Il-10, and IL-12 in patients with chronic liver failure were increased and the increase of IL-10 is secondary to elevation of IL-12.

## 5. Conclusion

Significant elevation of IL-10 and IL-12 with disease progression and transaminase values might be involved in chronic inflammation progression leading to HCC and their evaluation could be used as new biomarkers for HCC development.

## Figures and Tables

**Table 1:** Clinical, biochemical, and virological characteristics of the studied group.

	Chronic liver disease (*n* = 41)			NDC (*n* = 10)
	HCC	LC	PCH	
No (%)	15/41 (36.6%)	16/41 (39.0%)	10/41 (24.4%)	10/10 (100%)
Gender (M/F)	13/2	12/4	9/1	7/3
Age (years)	57.06 ± 12.58 (60.0)	46.06 ± 7.9 (45.0)	35.8 ± 5.2 (37.5)	35.6 ± 3.1 (35.5)
Albumin (g/dL)	4.22 ± 0.58 (4.3)	4.15 ± 0.467 (4.0)	4.5 ± 0.42 (4.6)	3.83 ± 0.29 (3.85)
T. protein (g/dL)	7.95 ± 0.603 (8.0)	7.6 ± 0.67 (7.8)	7.66 ± 0.49 (7.85)	7.22 ± 0.53 (7.15)
T. bilirubin (mg/dL)	1.69 ± 2.57 (1.2)	1.28 ± 1.54 (1.0)	0.99 ± 0.54 (0.9)	0.57 ± 0.21 (0.55)
AST (*μ*/mL)^*∗*^	92.8 ± 65.67 (67.0)	57.0 ± 26.6 (52.5)	34.8 ± 11.44 (30.5)	26.6 ± 5.5 (26.5)
ALT (*μ*/mL)^*∗∗*^	58.73 ± 36.86 (46.0)	49.0 ± 17.35 (49.0)	32.6 ± 14.8 (25.5)	27.7 ± 4.1 (27.5)
Proth. time (Sec)	14.3 ± 1.7 (14.0)	13.58 ± 4.66 (14.6)	13.2 ± 4.77 (13.3)	6.04 ± 0.568 (6.1)
Proth. conc. (%)	72.26 ± 13.78 (70.0)	70.375 ± 12.99 (69.0)	73.1 ± 19.0 (78.0)	258.4 ± 14.06 (256.0)

HCV infection				
Positive	11/15 (73.3%)	14/16 (87.5%)	8/10 (80%)	0/10 (0.0%)
Negative	4/15 (26.7%)	2/16 (12.5%)	2/10 (20%)	10/10 (100%)

Tumor site				
Right lobe	7/15 (46.7%)			
Left lobe	8/15 (53.3%)			

HCC grade				
Grade I	2/15 (13.3%)			
Grade II	4/15 (26.7%)			
Grade III	9/15 (60.0%)			

Child score				
A	3/15 (20.0%)			
B	6/15 (40.0%)			
C	6/15 (40.0%)			

Data expressed as mean ± SD (median).

Hepatocellular carcinoma (HCC), liver cirrhosis (LC), precirrhotic hepatitis (PCH), nondisease control (NDC).

^*∗*^Significant difference was detected between PCH and LC (*P* = 0.014) and HCC (*P* = 0.02).

^*∗∗*^Significant difference was detected between PCH and LC (*P* = 0.027) and HCC (*P* = 0.003).

**Table 2:** IL-10 and IL-12 in patients with different pathologies of the studied group.

	IL-10	*P* value	IL-12	*P* value
Chronic liver Disease				
HCC (*n* = 15)	16.13 ± 1.1	<0.0001	656.9 ± 19.11	<0.0001
LC (*n* = 16)	14.3 ± 0.765		505.8 ± 20.8	
PCH (*n* = 10)	6.47 ± 0.596		487.7 ± 17.2	
NDC (*n* = 10)	6.04 ± 0.568		258.4 ± 14.06	
HCC grading				
Grade I (*n* = 2)	16.6 ± 0.565	0.87	656.5 ± 34.648	0.9
Grade II (*n* = 4)	16.3 ± 0.72		659.75 ± 22.98	
Grade III (*n* = 9)	15.9 ± 1.33		655.7 ± 16.9	
Child score				
A (*n* = 3)	16.13 ± 0.305	0.7	664.3 ± 16.04	0.59
B (*n* = 6)	15.98 ± 1.15		658.3 ± 23.6	
C (*n* = 6)	16.28 ± 1.4		651.8 ± 19.1	

Data expressed as mean ± SD.

*P* value <0.05 is considered significant.

**Table 3:** IL-10 and IL-12 serum expression in chronic liver disease with HCV infection.

	+ve HCV-chronic liver disease^**∗∗∗**^				−ve HCV-chronic liver disease				NDC
	PCH	LC	HCC	Total	PCH	LC	HCC	Total	
IL-10 (ng/mL)^**∗**^	6.437 ± 0.518	14.49 ± 0.615	16.39 ± 1.015	13.169 ± 4.02	6.6 ± 1.13	13.0 ± 0.14	15.4 ± 1.16	12.61 ± 3.95	6.04 ± 0.568

IL-12 (ng/mL)^**∗∗**^	484.875 ± 16.23	501.86 ± 19.1	656.45 ± 17.1	549.27 ± 79.15	499.0 ± 22.63	533.5 ± 2.12	658.25 ± 27.035	587.3 ± 79.5	258.4 ± 14.06

^*∗*^IL-10 expression showed significant difference in HCV-infected (*P* < 0.0001) and noninfected patients (*P* < 0.003) compared to nondisease control.

^*∗∗*^IL-12 expression showed significant difference in HCV-infected and noninfected patients (*P* < 0.0001) compared to nondisease control.

^*∗∗∗*^+ve HCV-chronic liver disease shows that there is asymptotic significance (*P* < 0.0001) within groups (Kruskal-Wallis test) and the difference between groups was detected by Mann-Whitney showing that there is a significant (*P* < 0.0001) difference between each group and the other one.
